# EFFECT OF SIZE OF INTESTINAL DIVERSIONS IN OBESE PATIENTS WITH METABOLIC SYNDROME SUBMITTED TO GASTRIC BYPASS

**DOI:** 10.1590/0102-6720201600S10005

**Published:** 2016

**Authors:** Rafael Jacques RAMOS, Cláudio Corá MOTTIN, Letícia Biscaino ALVES, Daniela BENZANO, Alexandre Vontobel PADOIN

**Affiliations:** 1 Obesity and Metabolic Syndrome Center, Hospital São Lucas, Pontifical Catholic University of Rio Grande do Sul - PUCRS; 2 Program of Postgraduate Medicine and Health Sciences at PUCRS, Porto Alegre, RS, Brasil

**Keywords:** Bariatric surgery, Morbid obesity, Type 2 diabetes mellitus, Y-de-Roux Gastric Derivation, Lipid disorder, Metabolic syndrome

## Abstract

**Background:**

There is no consensus on the ideal size of intestinal loops in gastric bypass of bariatric surgeries.

**Aim:**

To evaluate the metabolic outcome of patients submitted to gastric bypass with alimentary and biliopancreatic loops of different sizes.

**Methods:**

Was conducted a retrospective cohort study in diabetic obese patients (BMI≥35 kg/m^2)^ with metabolic syndrome submitted to gastric bypass. The patients were divided into three groups according to the size of the intestinal loop: group 1, biliopancreatic limb 50 cm length and alimentary limb 100 cm length; group 2 , biliopancreatic limb 50 cm length and alimentary limb 150 cm length; and group 3, biliopancreatic limb 100 cm length and alimentary limb 150 cm length. The effect of gastric bypass with different sizes of intestinal loops in relation to the parameters that define metabolic syndrome was determined.

**Results:**

Sixty-three patients were evaluated, and they had a mean age of 44.7±9.4 years. All were diabetics, with 62 (98.4%) being hypertensive and 51 (82.2%) dyslipidemic. The three groups were homogeneous in relation to the variables. In 24 months, there was a remission of systemic arterial hypertension in 65% of patients in group 1, 62.5% in group 2 and 68.4% in group 3. Remission of diabetes occurred in 85% of patients in group 1, 83% in group 2 and 84% in group 3. There was no statistical difference in %LEW between the groups, and waist measurements decreased in a homogeneous way in all groups. The size of loops also had no influence on the improvement in dyslipidemia.

**Conclusion:**

Variation in size of intestinal loops does not appear to influence improvement in metabolic syndrome in this group of patients.

## RESUMO

**Racional:**

Não há consenso sobre o tamanho ideal das alças intestinais no bypass gástrico em Y-de-Roux em cirurgias bariátricas.

**Objetivo:**

Avaliar os desfechos metabólicos de pacientes submetidos ao bypass gástrico com alça intestinal alimentar e biliopancreática de tamanhos diferentes.

**Métodos:**

Realizou-se coorte retrospectiva em pacientes obesos (IMC≥35 kg/m^2)^ diabéticos com síndrome metabólica submetidos ao bypass gástrico em Y-de-Roux. Foram divididos em três grupos conforme a dimensão das alças intestinais: grupo 1, alça biliopancreática de 50 cm e alça alimentar de 100 cm; grupo 2, alça biliopancreática de 50 cm e alça alimentar de 150 cm e grupo 3, alça biliopancreática de 100 cm e alça alimentar de 150 cm. Foram avaliados os parâmetros que compõem a síndrome metabólica.

**Resultados:**

Incluíram-se 63 pacientes, com média de idade de 44.7±9.4 anos. Todos eram diabéticos, 62 (98.4%) hipertensos e 51 (82.2%) dislipidêmicos. Os três grupos eram homogêneos em relação às variáveis estudadas. Em 24 meses houve remissão da hipertensão arterial sistêmica em 65% do grupo 1, 62.5% no grupo 2 e 68.4% no grupo 3. A remissão do diabete melito tipo 2 ocorreu em 85% dos pacientes do grupo 1, 83% no grupo 2, e 84% no grupo 3. Não houve diferença estatística na porcentagem de perda do excesso de peso entre os grupos e as medidas da cintura abdominal reduziram de forma homogênea em todos os grupos. A dimensão das alças também não influenciou na melhora da dislipidemia.

**Conclusão:**

A variação da dimensão das alças intestinais não influenciou na melhora da síndrome metabólica neste grupo de pacientes.

## INTRODUCTION

The definition of better treatment for patients with morbid obesity and/or metabolic syndrome is a great challenge for bariatric surgeons and clinical investigators. The goal sought by all is to perform a safe and effective procedure, with the lowest rate of complications in the short term as well as long term.

In addition to the various types of surgery for the treatment of these patients, the existing variations within each technique are diverse. Gastric bypass is one of the most utilized surgeries in the world for the treatment of these patients, but the literature still lacks in the definition of the ideal sizes of intestinal loops.

Considering that many obese patients with indication for bariatric surgery are diabetics and have metabolic syndrome and that the surgical techniques with longer intestinal diversions are potentially more deleterious in the uptake of nutrients^6^ we carried out a study to evaluate the metabolic outcomes of these patients submitted to gastric bypass with alimentary and biliopancreatic loops of different sizes.

## METHODS

The project was approved by the Scientific Committee and the Committee of Ethics and Research of the institution (#11/05596).

Was conducted a retrospective cohort study in obese patients (BMI ≥35 kg/m^2^) diabetics with metabolic syndrome submitted to gastric bypass in a tertiary referral center for the treatment of such patients. The study included obese patients meeting the criteria of diabetes and metabolic syndrome defined by the International Diabetes Federation (IDF)^30^, who had a minimum follow-up of two years. Regular physical activity practitioners were excluded (150 min/week), those who had complications (fistula, internal hernia, intestinal obstruction) or requiring re-intervention , those who underwent cosmetic surgery or reconstructive abdomen, malignancy history (before or after the operation) and the user of chronic corticosteroids were also excluded.

The variables were measured in the preoperative period and following postoperative times: 3, 6, 12 and 24 months. The data collected were anthropometric measurements, blood pressure levels and the results of laboratory tests. Also identified were the patients who continued using medications in the postoperative period (antihypertensives, oral antidiabetics and/or insulin and antilipemics). The anthropometric data evaluated were weight, height and waist circumference. The serum laboratory tests evaluated were fasting glycemia, total cholesterol, high-density lipoprotein cholesterol (HDL-C) and triglycerides.

The criteria utilized for the identification of patients with metabolic syndrome were based on the classification of the IDF. The waist circumference cutoff was ≥90 cm in men and ≥80 cm in women. Patients were considered hypertensive if systolic (SBP) and diastolic (DBP) blood pressure was ≥130/85 mmHg or if using antihypertensive medication. Patients were considered dyslipidemic if serum triglycerides ≥150 mg/dl and/or HDL-C <50 mg/dl in women and <40 mg/dl in men, or if using antilipemic medication. All patients in the sample were diabetics according to the criteria of the American Association of Diabetes (ADA)^18^.

The patients were divided into three groups according to the size of intestinal loops: group 1, biliopancreatic loop of 50 cm and alimentary loop of 100 cm; group 2, biliopancreatic loop of 50 cm and alimentary loop of 150 cm; and group 3, biliopancreatic loop of 100 cm and alimentary loop of 150 cm. 

### Statistical analysis

The quantitative variables were expressed as means and standard deviations when their distribution was symmetric and compared between the groups by analysis of variance (ANOVA). These variables were compared within groups by the Student t-test for paired samples. The differences in the variation of the parameters over time between the groups were evaluated utilizing ANOVA for repeated measurements. The categorical variables were expressed as frequencies and percentages and compared by the chi-square test. In all cases, differences were considered significant if p ≤0.05.

## RESULTS

Sixty-three patients were evaluated, where 48 (76%) were females. The mean age was 44.7±9.4 years. All patients were diabetics, with 62 (98.4%) being hypertensive and 51 (82.2%) dyslipidemic (Table 1). The results obtained in each assessment are presented in Table 2.

**TABLE 1:** Characteristics of sample

	Group 1 (n=20)	Group 2 (n=24)	Group 3 (n= 19)	p
Age (years)	46.2±9.8	43.7±7.7	44.3±11.2	0.692
Gender, female	15 (75%)	18 (75%)	15 (79%)	0.945
Weight (kg)	121.2±21.9	129.2±22.3	125.77±17.3	0.451
BMI* (kg/m2)	45.9± 7.4	46.8± 6.7	46.9± 5.8	0.877
Waist (cm)	129±17	134±14	131±11	0.523
SAH**	19 (95%)	24 (100%)	19 (100%)	0.335
DM2***	20 (100%)	24 (100%)	19 (100%)	1.000
Dyslipidemia	15 (75%)	21 (87%)	15 (79%)	0.555

*BMI=kg/m^2^; **SAH=systemic arterial hypertension; *** DM2=type 2 diabetes mellitus

**TABLE 2:**
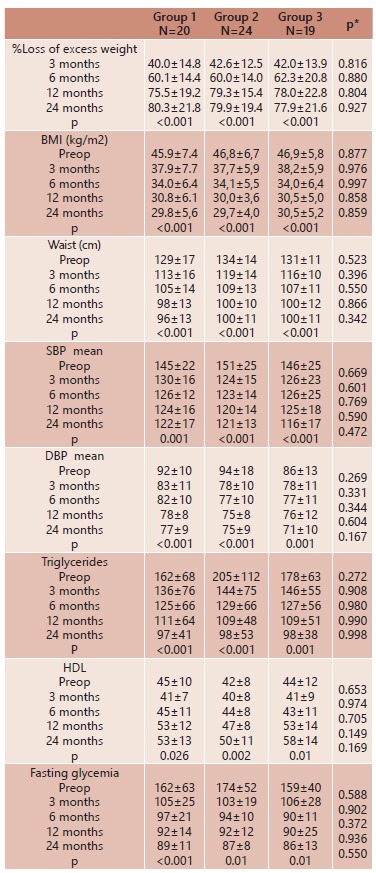
Characteristics and results of the groups

### Loss of excess weight, BMI

**FIGURE 1:**
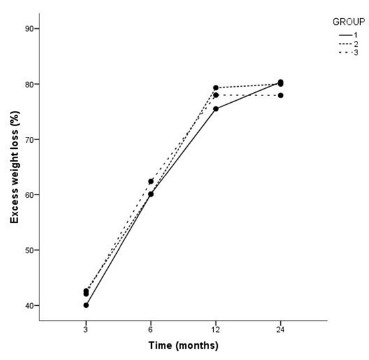
Mean loss of excess weight (%) in groups 1, 2 and 3 at 3, 6, 12 and 24 months

The percent loss of excess weight (%LEW) is presented in Table 2, showing that there was no significant difference between the three groups, p>0.05. The patients of the three groups showed similar results with respect to BMI at all times evaluated, p<0.001. Figure 1 displays the mean %LEW for the three groups at times 3, 6, 12 and 24 months.

### Waist circumference 

Waist measurements decreased in a homogeneous way in all groups evaluated. Despite a statistically significant reduction (p<0.001) in relation to the data before surgery, only 3% (n=2) attained measurements below the criteria suggested by the IDF. In group 1, 10% (n=2) reached the criteria recommended by the IDF, which was not observed by any patient in groups 2 and 3. There was no statistical difference with respect to waist measurement between the three groups at any moment evaluated, p >0.05. 

### Systemic arterial hypertension (SAH)

In the evaluation of arterial hypertension, 63.1% of patients in group 1 had controlled blood pressure at 24 months, while 47.3% showed control at three months, without medication. In group 2, 62.5% of hypertensives showed controlled SAH at 24 months without medication, but 20% still had uncontrolled SAH at this time, even with the use of antihypertensive drugs. In this group, 37.5% of patients showed control of SAH by the third month. In group 3, 68.4% had controlled SAH at 24 months, where 42.1% achieved this control already by the third month after surgery. There was no statistical difference with respect to resolution of SAH between the three groups at any moment evaluated, p >0.05.

### Triglycerides

The reduction in triglyceride levels was similar in the three groups. At 12 months, there was a decrease in triglycerides of 39%, and at 24 months, a decrease of 46%. Triglycerides were decreased and remained below 150 mg/dL in 73% of all patients, starting at 12 months (group 1: 80%; group 2: 64.2%; group 3: 76.9%), where levels were stable for up to 24 months. In group 1, mean reduction in triglycerides at 24 months was 65.5 mg/dl, whereas 106.6 mg/dl in group 2 and 79.9 mg/dl in group 3. There was no statistical difference with regard to the measurement of serum between the three groups at any moment evaluated, p>0.05. 

### HDL 

In group 1, 86.6% of the dyslipidemic patients showed a serum HDL-C level lower than the reference value of the IDF; in groups 2 and 3, the proportions were 79.1 and 60%, respectively. At three months, there was a reduction in HDL-C levels in the three groups, followed by an elevation in the next periods up to 24 months. Despite a discrete reduction in HDL-C in the period of 12 to 24 months in group 1, the mean HDL-C level remained about 53 mg/dl. The mean increase in HDL-C for the three groups was 7.6 mg/dl at 24 months. There was no statistical difference with respect to serum HDL-C between the three groups at any moment evaluated, p>0.05.

### DM2

**FIGURE 2:**
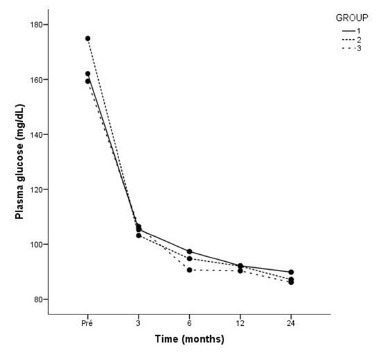
Mean fasting glycemia levels (mg/dl) in groups 1, 2 and 3 in preoperative period and at 3, 6, 12 and 24 months.

In group 1, 85% of patients had DM2 controlled without medication already in the third month after surgery. After 24 months, only one patient needed medication to control DM2. In group 2, 83% of patients had DM2 controlled without medication in the third month after surgery. At 24 months, only one of the patients needed an oral hypoglycemic agent to control DM2. In group 3, 84% controlled DM2 by the third month, remaining so on the 24^th^ month. There was no statistical difference with respect to resolution of DM2 between the three groups at any moment evaluated (p>0.05). Figure 2 shows the mean fasting glycemia (mg/dl) at the different evaluation times: in the preoperative period and at 3, 6, 12 and 24 months.

## DISCUSSION

In this study, was observed a curve for %LEW similar to those seen in the literature^5^ ^,^ ^11^ ^,^ ^24^ ^,^ ^25^ and also that the different sizes of intestinal loops did not influence this variable. Two retrospective studies by Christou^6^and Feng^12^ found no statistical difference in relation to weight loss and %LEW in a follow-up of 60 months and 12 months, respectively, when comparing different sizes of intestinal loops. A prospective study by Sarhan et al.^25^ also showed no statistical difference in loss of excess weight and in weight regain in a follow-up of 24 months in patients with BMI >50 kg/m^2 8^. There are three randomized studies^5^ ^,^ ^16^ ^,^ ^24^ with similar results as this one. Choban^5^ evaluated the effect of the size of the alimentary loop in 128 patients. There was no statistically difference in 24 and 36 months. Pinheiro^24^ noted that the loss of excess weight in patients with a BMI ≥50 kg/m^2^ was faster in the group with the highest intestinal loop, but was similar in the groups evaluated in 48 months. Inabnet^16^ also showed no statistically difference in weight loss and %EWL in patients with intestinal loops of different sizes in 24 months. However, Brolin^3^ in their prospective randomized study found statistically significant weight loss in the long bypass in 24 and 36 months (p≤0.02). Ciovica^7^ also showed statistically difference in mean BMI and the final average %EWL in greater alimentary loop, suggesting that alimentary loop should be larger than 100 cm

### Waist circumference 

In this study, there was a decrease in waist circumference in the thre groups, and there was no statistically significant difference over time in relation to size of the intestinal loops. However, the mean waist circumference stayed above the values recommended by the IDF in all the periods evaluated. It should be pointed out that no patient was submitted to aesthetic or reparatory surgery in the abdomen and that part of this measurement could probably be attributed to lipodystrophy and not to visceral adiposity. Despite being one of the criteria of the IDF, waist circumference is an indirect inference of visceral adiposity and does not differentiate lean mass from adipose tissue and nor does it evaluate excess skin and subcutaneous tissue^17^.

Our results are similar to those found in other studies^17^ ^,^ ^23^, although there was a difference in size of intestinal loops and time of follow-up. In the study of Inge^17^ (biliopancreatic loop 10-15 cm and alimentary loop 100-150 cm ) there was a reduction in waist circumference of 144.5±8.7 cm to 107.4±8.4 cm in 12 months. In a randomizad study^23^ (biliopancreatic loop 30 cm and alimentary loop 75 cm) there was an average reduction in waist circumference of nearly 30 cm in 12 months.

### Blood pressure 

 Our results showed an early reduction in blood pressure. Evaluating all patients, there was an improvement in blood pressure in 91.8% of patients, although 35.5% (n= 22) continued to use antihypertensives, and a resolution of 67.2% SAH, at 24 months. Similar results are found in some studies^1,18,23^ with similar-sized intestinal loops as utilized in this research, but there are differences in relation to time of follow-up. In some studies, the sizes of the intestinal loops are not specified^16^ ^-^ ^19^, but the results are similar to those of our study, such as in the meta-analysis of Buchwald^4^ with a resolution of arterial pressure of 62%. 

Fernstrom^13^ (biliopancreatic loop of 50 cm and alimentary loop 150-250 cm) had a resolution of 50 % of the pressure at 18 months . Her assessment was not stratified by intestinal loop size. Ahmed^1^ (biliopancreatic loop 30 cm and alimentary loop 150 cm) obtained better results with resolution of hypertension of 88% in 12 months , but were not accounted patients lost follow-up. Dallal^8^ (biliopancreatic loop 40 cm and alimentary loop 75-150 cm) obtained from the pressure resolution 44.2% in 12 months. Other studies^15^ ^,^ ^29^ evaluated only the size of the alimentary loop, with results also similar to those presented here.

### Dyslipidemia

Although HDL-C decreased in the first three months, there was improvement in dyslipidemia in the three groups evaluated in our study. There was a greater reduction in triglycerides in the first three months, in the three groups, and an elevation in HDL-C after this period. Probably the worsening of HDL-C was related to the restricted diet on which patients were kept in the initial postoperative period, and this was also described in other studies^19^ ^,^ ^21^.

Gastric bypass has been shown to be effective in improving the lipid profile as evidenced in the SOS study^28^, where remission of hypertriglyceridemia occurred in 62% of patients at 24 months and normalization of HDL-C levels in 76%. There are few studies comparing the size of intestinal loops with lipid profile. Studies with a variation in biliopancreatic loop of 50-75 cm and alimentary loop of 75-250 cm have reported similar results as ours^8^ ^,^ ^24^, although with variation in time of follow-up. This favorable result is also identified in studies describing only the sizes of the alimentary loop (variation of 75-250 cm)^14^ ^,^ ^21^. The improvement of the lipid profile seems to maintain long-term. In the study of Jamal^19^ with six- year follow-up, in 76% of patients there was a reduction of triglycerides to the desired levels in six months and remained until the end of the study period. There was also an increase in HDL. These results are similar to Brolin^2^ that also demonstrated the permanence of satisfactory result in five years even with regained weight or insufficient weight loss

### Diabetes

Hyperglycemia and diabetes are associated with obesity, but improvement in glycemic levels occurs soon after the surgical procedure before a significant reduction in weight^9^ ^,^ ^10^. In our study, remission of diabetes was seen in 92.0% at 24 months, where in the first three months, the remission rate was 82.5%, and the mean BMI was 37.9±0.2 in this period. This result is similar to that of other studies^4,22^ and reinforces the action of the incretin phenomenon in glycemic control. There are few papers^20^ ^,^ ^22^ ^,^ ^24^ ^,^ ^26^ ^,^ ^29^ assessing the effect of the size of the bowel gastric bypass in relation to diabetes.There is evidence of improvement in diabetes or remission of the disease in various high-impact publications^4^ ^,^ ^28^. In the meta-analysis of Buchwald^4^ involving 136 studies and more than 22,000 patients, remission of diabetes was 83.7%. The multicenter study SOS in a follow-up of 10 years described a remission rate of 72%^27^ ^,^ ^28^. Some relate the size of the bowel with the DM2^24 , 26, 29^. When we compared the studies with a variation of biliopancreatic loop of 30-75 cm and a variation of alimentary loop of 75-200 cm^26^, was observed remission in 42-87% in the period of 12-18 months and a remission rate of 62-87% in the period of 24 months. In some studies it was observed remission of DM2 long term^29^ associated with biliopancreatic loop 40-60 cm and alimentary loop 60-150cm. The remission in 5-7 years was 86 % and in 14 years was 91%. Some authors^24^ show difference in control DM2 compared two groups with different sizes of loops (group1 , biliopancreatic loop 50 cm and alimentary 50 cm; group 2 , biliopancreatic loop 100 cm and alimentary 250 cm). The longer intestinal deviations have better control of DM2 (p<0,05).

Our study has some limitations in relation to the assessment of diabetes. Glycosylated hemoglobin was not determimed in the period of three to 24 months because it is not a parameter utilized for the definition of metabolic syndrome, according to the IDF^30^. In our study, was utilized only the variables that define metabolic syndrome. Also, the quantity of medications taken by each patient was not determined, only whether an oral antidiabetic agent and/or insulin was utilized. The time between the diagnosis of diabetes and the surgical procedure is a factor that affects the remission of diabetes, but that was not evaluated in the present study; was demonstrated an improvement in the control of obesity and the comorbidities studied, but contradicting our hypothesis that a surgery with longer intestinal loops would have a more potent effect, we did not see a difference in the results of patients in the three groups evaluated. Despite the small number of patients, the samples were homogeneous and the patients were evaluated in a systematic way by the same team, and the methods of measurement were identical among the three groups.

## CONCLUSION

Variation in the size of the bowel does not seem to influence the improvement of metabolic syndrome in patients undergoing gastric bypass.
